# Why do patients with Parkinson’s disease fall? A cross-sectional analysis of possible causes of falls

**DOI:** 10.1038/npjparkd.2015.11

**Published:** 2015-06-11

**Authors:** Anette Schrag, Mahbuba Choudhury, Diego Kaski, David A Gallagher

**Affiliations:** 1 Department of Clinical Neurosciences, Institute of Neurology, Royal Free Campus, University College London, London, UK

## Abstract

**Background:**

Falls in Parkinson’s disease (PD) are associated with significant injury, disability, hospitalization, and reduced quality of life.

**Aims:**

To identify modifiable medical causes of falls in a cohort of PD patients.

**Methods:**

Eighty seven PD patients were interviewed and examined using validated scales assessing motor and nonmotor aspects of PD, comorbidities and medication use. The frequency of falls in the last month was the primary outcome measure. Falls were hypothesized to be associated with increasing age, advanced motor severity, particularly axial features (e.g., freezing and postural instability), and dyskinesia. Nonmotor features hypothesized to be associated with falls included; cognitive impairment, psychosis, sleep disorders, cardiovascular dysfunction, and ophthalmological and medical comorbidities.

**Results:**

Fallers had longer disease duration, higher Levodopa-equivalent doses, greater ‘On’ time with dyskinesia (all *P*<0.005), and higher scores on some Movement Disorder Society-Unified Parkinson’s Disease Rating Scale items, particularly axial scores. However, patients with falls did not differ from non-fallers in age or overall motor UPDRS scores. Severity of psychosis, executive cognitive impairment, autonomic (particularly cardiovascular) dysfunction and sleep disturbances (particularly REM sleep behavioral disorder) were significantly associated with falls (all *P*<0.005). Fallers more frequently reported use of antidepressants (both tricyclics and SSRIs) and neuroleptics (*P*<0.001), but not hypnotics. There was no difference in medical comorbidities, ophthalmological assessments, fatigue, and apathy scores between the groups. In logistic regression analysis, cardiovascular dysfunction, antidepressant use, and REM sleep behavioral disorder were significantly associated with falls.

**Conclusions:**

The causes of falls in PD are multifactorial and extend beyond motor impairment and dyskinesia; addressing these in patients already treated with dopaminergic medications has the potential to improve this important complication of PD.

## Introduction

Falls in Parkinson’s disease (PD) can lead to injury and hospital admissions, and result in increased disability, fear of falling, low mood, and reduced quality of life.^1^ Despite being recognized as a difficult management aspect of advanced disease,^2^ correlation with disease duration and motor severity is only moderate.^3^ Although impairment of postural stability leading to falls is one of the core features of PD,^4^ it is likely that other causative factors have an important role and addressing these may reduce the risk of falling. Several studies have examined possible ways of predicting falls, with previous occurrence of falls being one of the main predictors^5,6^ and several motor factors including measures assessing postural stability,^7,8^ abnormal posture,^9^ freezing of gait,^7,9,10^ impairment of rapid alternating movement,^3^ and dyskinesia^10^ identified. Cognitive impairment, including measures of global^11^ and executive function^9,12^ have also been associated with falls in PD. Other nonmotor features including sleep, particularly rapid eye movement (REM) sleep behavioral disorder (RBD),^13,14^ autonomic symptoms,^8^ depression,^15^ cardiovascular and musculoskeletal comorbidity,^12^ and medication use, such as hypnotics and antidepressants,^12^ have also been implicated.

Amongst Parkinsonian features, whilst axial motor features such as posture, postural stability, and freezing of gait have been associated with falls in PD, the relationship with overall motor severity is complex^6^ and there is little available detailed quantitative information on individual axial and nonaxial items and their relative potential to cause falls. Also, most studies have generally focused on motor or nonmotor features independently, rather than the coexistent and complex inter-relationship of these factors and how this impacts on falling. Such information would be useful to understand the mechanisms of falls and guide management strategies to prevent them.

On the basis of previous studies, we hypothesized that age and disease severity, prominent axial motor features, and presence of dyskinesia could directly cause falls in PD. In addition, given the growing evidence linking falls with nonmotor features, we assessed autonomic symptoms, particularly cardiovascular function, general and executive cognitive impairment, sleep disturbance, mood, medical comorbidities and medications on the risk of falls. Finally, it is not known whether reduced vision or visual perception impacts on the propensity to fall and this was further investigated in a subset of patients.

## Materials and methods

Consecutive patients fulfilling UK Brain Bank criteria for PD^4^ who participated in a previous study^16^ were recruited from PD outpatient clinics. In brief, subjects underwent face-to-face interview, comprising clinical examination and physician-administered questionnaires and were given further questionnaires to complete at home. A subgroup of patients, who consented to an additional appointment, also had a full ophthalmological assessment.

The main outcome variable was a question to the patient on the frequency of falls in the last month. This stratified the number of falls in the last month, into four distinct categories; never falling, falling once, twice and three times, or more. This was chosen as the assessment was retrospective and recall beyond 1 month is unlikely to be reliable. As the majority of patients had not had falls, patients were classified into non-fallers (never falling) and fallers (falling at least once in the last month).

Relevant other assessments that were included in this study were the Movement Disorder Society (MDS)-Unified Parkinson’s Disease Rating Scale (MDS-UPDRS);^17^ percentage of time ‘On’, ‘On’ with dyskinesia and ‘Off’ time; Scales for Outcome in Parkinson’s (SCOPA) cognitive scale (SCOPA-COG),^18^ SCOPA-autonomic scale (SCOPA-AUT);^19^ Pittsburgh Sleep Quality Index (PSQI);^20^ Epworth Sleepiness Scale;^21^ International Classification of Sleep Disorders, Revised (ICSD-R) diagnostic criteria for REM sleep behavioral disorder;^22^ Parkinson Psychosis Rating Scale;^23^ Fatigue Severity Scale;^24^ Lille Apathy Rating Scale (LARS);^25^ Hospital Anxiety and Depression Scale;^26^ and checklist of a range of comorbidities and medications. A subgroup of 46 patients also had ophthalmological measures including logarithm of minimal angle of resolution (logMAR) visual acuity testing,^27^ Goldmann kinetic perimetry, mean peripheral field diameter expressed as mean radial degrees (MRD),^28^ retinal photography, descriptive assessment of cataract presence, location (nuclear, cortical, and subcapsular), and degree of opacity and 76 had a test for visual object recognition, the Birmingham Object Recognition Battery.^29^ Ethical approval was obtained from the local research ethics committees and informed consent was obtained from all participants.

### Statistical analysis

Statistical analysis was performed using SPSS version 20.0 (SPSS Inc., Chicago, IL, USA). Data were visually and statistically assessed for normality using histograms and the F-test function. Nonparametric data were expressed as a median and a range. In this analysis, only variables that were *a priori* hypothesized to potentially be causative for falls were compared between fallers and non-fallers. Scales with overlapping content were avoided. Scale scores were compared using two-sided Mann–Whitney and categorical variables using *χ* ^2^-tests, with a significance level of *P*<0.01 to adjust for multiple comparisons. A forward-stepping logistic regression model was carried out entering variables associated with falls in univariate analysis (*P*<0.01), using subscale scores rather than total scores where applicable. As the MDS-UPDRS includes items on the majority of motor and nonmotor aspects of PD and provides more detailed information on motor aspects, we performed a separate logistic regression analysis of falls on all MDS-UPDRS items with a significance level of *P*<0.05.

## Results

Twenty-seven of 87 (31%) of participants had experienced falls in the last month, and of those, 16 had 1, 6 had 2, and 5 had 3 or more falls. There was no difference in age or sex (Table 1). Those who reported falls had significantly longer disease duration (median 10.8 vs. 4.1 years, *P*<0.01), higher Levodopa Equivalent Unit doses (*P*<0.01), as well as MDS-UPDRS part I (Nonmotor Experiences of Daily Living), part II (Motor Experiences of Daily Living), and part IV (Motor Complications) scores, reflecting more advanced disease. Those who had fallen reported a higher proportion of the day with dyskinesia and reduced ‘On’ without dyskinesia episodes compared with non-fallers. However, there was no significant difference between the groups on MDS-UPDRS part III (Motor Examination).

Fallers performed significantly worse on executive cognitive function tests (SCOPA-COG), psychosis (PPRS) and sleep (PSQI) scores and presence of RBD was significantly higher in fallers. However, there was no significant difference between the two groups for daytime sleepiness (ESS), fatigue (FSS), apathy (LARS), anxiety or depression scores (HADS).

Patients with falls had significantly more autonomic symptoms (SCOPA-AUT), particularly greater cardiovascular, pupillomotor, and gastrointestinal autonomic symptoms were reported.

In terms of dopaminergic or other medication use (Table 2), fallers were significantly more likely to be on antidepressants (both selective serotonin reuptake inhibitors and tricyclics) and neuroleptics (Quetiapine only). No significant difference was found for sleep- and other medication or for any comorbidities (although associations with self-reported depression and psychosis approached significance *P*<0.05).

There was no difference in visual acuity, visual fields, and any of the other ophthalmological parameters between fallers and non-fallers.

### Logistic regression analysis

Variables associated in univariate analysis at *P*<0.01 significance (antidepressant use, neuroleptic use, LEU dose, percent of daytime in ‘On’ with dyskinesias, percent of daytime in ‘On’ without dyskinesias, SCOPA-COG-executive function, SCOPA-AUT-Cardiovascular, Gastrointestinal, and Pupillomotor scores, PPRS, RBD, total PSQI score, and duration since diagnosis) were included in a forward stepwise conditional logistic regression. The logistic regression model identified the use of antidepressants, presence of RBD and cardiovascular autonomic dysfunction as the main factors associated with falling in the last month (Table 3).

### MDS-UPDRS items

**Figure 1:**
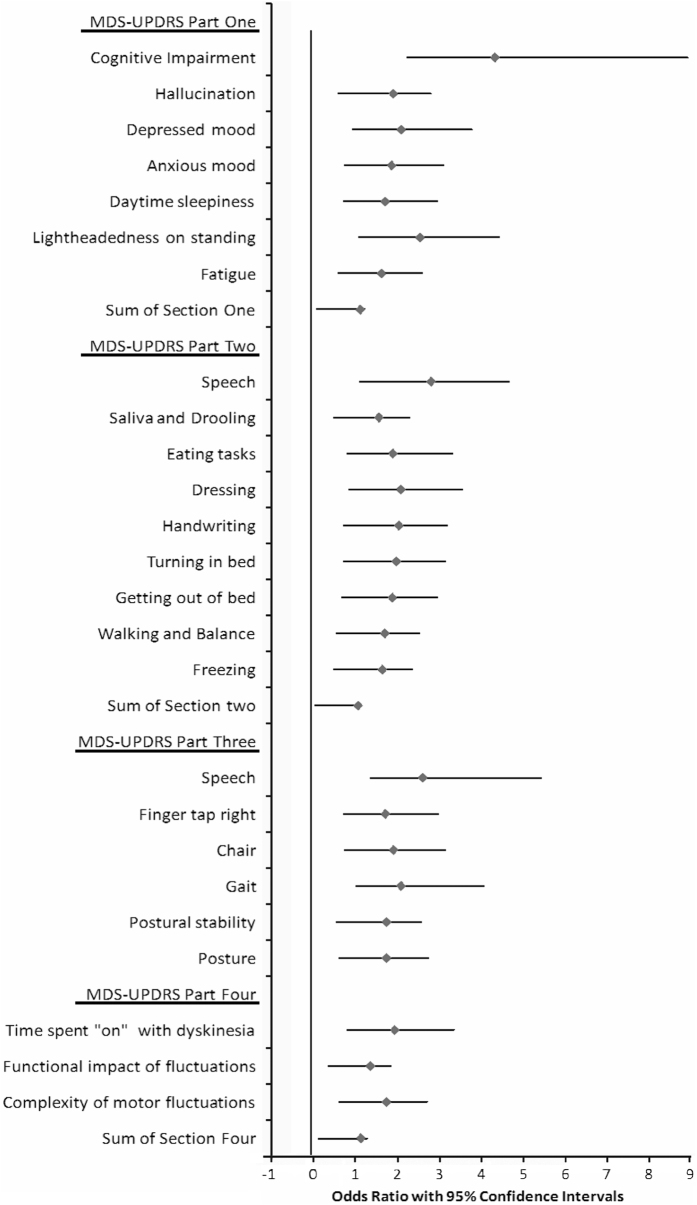
Odds ratios with 95% confidence intervals for individual Movement Disorder Society (MDS)-Unified Parkinson’s Disease Rating Scale (MDS-UPDRS) items significantly associated with risk of falling in univariate logistic regression.

Amongst individual MDS-UPDRS items (Figure 1), occurrence of falls was associated with higher scores in Part I for physician-assessed items of cognition, hallucinations, anxiety, and depression and also self-reported light-headedness on standing, fatigue, and daytime sleepiness. In part II, significantly associated self-reported items included difficulties with speech, handwriting, turning in bed, getting out of bed, freezing, walking and balance, dressing, saliva and drooling, and eating tasks. In Part III, falls were associated with difficulties with speech and finger tapping, and with the predominantly axial features arising from a chair, gait, posture and postural instability, and in Part IV with the proportion of time spent ‘On’ with dyskinesia, functional impact of motor fluctuations and complexity of motor fluctuations.

## Discussion

The analysis of potential causes of falls in PD showed that nonmotor features of PD, particularly RBD, autonomic impairment and antidepressant medication use, are associated with falls over and above what is explained by motor severity, disease duration or dyskinesias in clinic populations of patients with PD.

### Motor variables

As expected, those with falls had longer disease duration and higher levodopa dose, and greater axial symptoms, particularly stooped posture, postural instability, impaired gait, including freezing episodes (by history) and inability to rise from a chair. Of note, we did not find any association between falls and the overall motor severity as assessed on the motor MDS-UPDRS, similar to what has been reported previously.^30^ The MDS-UPDRS also captures tremor and limb akinesia, which are less likely to affect postural stability and may be more responsive to dopaminergic medication. In the literature, disease severity measurements have also not been shown to be a good predictor of falls. However in a meta-analysis of six prospective studies of falling in PD^6^ there was a complex U-shaped relationship, with the risk of falling increasing as disease severity increased, but remained at this level thereafter and there was tendency to taper off towards later disease stages. This may be because those with more advanced disease attempt to walk less often. In addition, motor fluctuations were associated with falls, as were freezing episodes by history but not on examination, suggesting that axial features such as freezing during off-periods contribute to falls but may not be obvious during the consultation. Dyskinesias were also associated with increased falling in this study, as previously reported by others.^10^

### Nonmotor variables

Fallers also scored significantly worse on scales for a number of nonmotor features, particularly executive cognitive function, autonomic symptoms, psychosis, and sleep disturbances. Consistent with this, higher scores on the MDS-UPDRS part I items on cognition and hallucinations were associated with falls. One of the strongest associated factors was sleep disturbance (particularly RBD), which may suggest that these complications of PD arise from the same pathology as, or alternatively sleep disturbances independently contribute to, falls and are potentially amenable to treatment. REM sleep behavior disorder has previously been linked to falls in PD.^31^ Several studies also previously found that falls were significantly associated with cognitive impairment (in particular attention and executive dysfunction),^9,11,12,32,33^ psychosis^34^ and cardiovascular dysfunction^3,35^ and are often under-recognized.

### Potential underlying pathophysiological mechanisms

Although falls are one of the key features of advanced PD,^8^ and dopaminergic medications can improve balance impairment in PD,^36^ postural instability in PD is comparatively less responsive to levodopa therapy than limb akinesia or tremor to levodopa therapy, and it is likely that other pathophysiological mechanisms are involved in gait disturbance in these patients.^37,38^ It has been postulated that noradrenergic deficits such as in the locus coeruleus^37^ and cholinergic pathways like the pedunculopontine have an important role.^38^ In support of the noradrenergic hypothesis, a large randomized controlled study using methylphenidate in advanced PD patients, improvements were observed for gait bradykinesia and freezing of gait.^39^ In animal models, in rats, dual cholinergic-dopaminergic lesions were found to result in falls more frequently than those with either cholinergic or dopaminergic lesions.^40^

The findings of this study confirm from a clinical point of view that falls are associated with nonmotor features related to non-dopaminergic dysfunction, i.e., autonomic dysfunction, psychosis, sleep disorders, and cognitive impairment. All of these are thought to arise at least in part from deficits in the noradrenergic and cholinergic pathways with complex interaction of several neurotransmitter systems.^41,42^

This association of falls with nondopaminergically determined nonmotor features raises important considerations for therapy. For example, recently it has been reported that donepezil reduced falls in PD patients.^43^ Whether this is related to cognitive improvements or direct cholinergic effects on gait and balance is unknown. It has also been reported that task-specific cognitive training improved gait velocity, gait symmetry, and obstacle negotiation.^44–46^ In addition, in patients undergoing Deep Brain Stimulation of the cholinergic pedunculopontine nucleus-targeted, which has also been implicated in the pathophysiology of gait disturbance in PD, there was significant improvement in nocturnal sleep and reduction of daytime hypersomnolence.^47^

### Comorbidities

There was no association with other comorbidities as has been reported previously or with ophthalmological factors. However, comorbidities and visual dysfunction^48^ were not common in our group and this does therefore not exclude that individual patients with visual or other comorbidities are more prone to falls as a result of visual impairment, which has been reported in the literature.^12^

### Medication

Antidepressant and antipsychotic use were associated with falls, which, as reported previously, may be due to their sedating effects^5,12^ Diagnosis and treatment of depression in PD is complex and mood disorders in the context of neurodegeneration may not respond in the same way to medication developed for endogenous depression. Evidence for safety and efficacy of individual antidepressants in PD is limited because of few large double-blind trials and methodological considerations.^49,50^ Similarly, the benefit of psychotic medication in PD has not been established for the majority of commonly used agents, including quetiapine the antipsychotic used in patients in this study.^50,51^ Our study further emphasizes that psychotropic medication can be associated with adverse effects, including falls, and caution should be exercised in using these drugs particularly if efficacy has not been confirmed in PD.

### Limitations of study

Falls information on our patients was recorded retrospectively. Thus, number of falls may be underestimated, and the study could only examine associations rather than causality. Prospective studies need to examine causation of falls and should optimally be over longer periods of time. We also relied on patient report on comorbidities and clinical examination and did not exclude comorbidities such as vascular disorders or vestibular disorders with further specific testing. The relative prevalence of these comorbidities in PD and controls and their contribution to the occurrence of falls may be area of further research. Nevertheless, this broad clinical assessment of potential causes of falls highlights multiple factors associated with falls that can potentially be addressed in these patients.

## Conclusion

The causes of falls in PD are multifactorial and extend beyond progressive motor impairment and dyskinesia. Given the limited levodopa responsiveness of gait dysfunction and imbalance, future therapies should also address cognitive and other nonmotor factors, simultaneously addressing dopaminergic, cholinergic, and noradrenergic pathways, and prescription of psychotropic medications should be reviewed critically to avoid exacerbation of falls.

## Figures and Tables

**Table 1:** Demographics and motor and nonmotor scale scores between fallers and non-fallers

	*Median (range) or Number (%)*		P*-value* a
	*Fallers (*n*=27)*	*Non-fallers (*n*=60)*	
*Demographics*			
Age (years)	70 (58–83)	67.5 (44–86)	0.44
Disease duration (years)	10.8 (0.1–29.1)	4.1 (0–22.8)	0.005
			
*Motor features*			
MDS-UPDRS part III	36 (12–65)	34 (9–70)	0.24
Dyskinesia (% daytime)	6 (0–75)	0 (0–53)	0.005
‘On’ without dyskinesia (%)	76 (12–100)	100 (7–100)	0.006
‘Off’ time (%)	12 (0–56)	0 (0–54)	0.04
Hoehn and Yahr stage	3 (1–4)	2 (2–5)	0.018
			
*Cognition*			
SCOPA-COG-total	23 (9–32)	26 (6–39)	0.02
SCOPA-COG-memory	8 (3–13)	9 (1–18)	0.07
SCOPA-COG-attention	4 (2–4)	4 (0–4)	0.24
SCOPA-COG-executive	8 (2–12)	9 (2–12)	0.003
SCOPA-COG-visuospatial	4 (1–5)	4 (0–5)	0.25
			
*Sleep*			
PSQI	7 (2–19)	5 (1–18)	0.007
Epworth Sleepiness Score	11 (1–20)	5.5 (0–20)	0.04
Presence of RBD	13/27 (48%)	11/60 (18%)	0.004
			
*Psychosis, apathy, fatigue, and depression*			
PPRS	9 (6–14)	6 (6–15)	<0.001
Lille Apathy Rating Scale	−25 (−35 to −3)	−26.5 (−35 to –2)	0.30
Fatigue Severity Scale	4.6 (1.8–7)	4.0 (0–7)	0.06
HADS anxiety	7 (1–15)	4.5 (0–16)	0.06
HADS depression	6 (2–12)	4.5 (0–18)	0.11
			
*Autonomic function*			
SCOPA-AUT-total	18 (11–36)	10 (3–35)	<0.001
SCOPA-AUT-gastrointestinal	4 (1–8)	2 (0–10)	<0.004
SCOPA-AUT-urinary	7 (1–15)	4 (0–16)	0.02
SCOPA-AUT-cardiovascular	1 (0–6)	0 (0–3)	0.001
SCOPA-AUT-thermoregulatory	2 (0–11)	1 (0–10)	0.07
SCOPA-AUT-pupillomotor	1 (0–3)	0 (0–3)	0.001
SCOPA-AUT-sexual	3 (0–6)	2 (0–6)	0.14

Abbreviations: HADS, Hospital Anxiety and Depression Scale; MDS-UPDRS, Movement Disorder Society-Unified Parkinson's Disease Rating Scale; PPRS, Parkinson Psychosis Rating Scale; PSQI, Pittsburgh Sleep Quality Index; RBD, REM sleep behavioral disorder; SCOPA-AUT, Scales For Outcomes in Parkinson's Disease Autonomic questionnaire; SCOPA-COG, Scales For Outcomes in Parkinson's Disease Cognition.

a Mann–Whitney or *χ* ^2^-test.

**Table 2:** Antiparkinsonian and other medication use

	*Median (range) or number (%)*		*Significance *P*-value*
	*Fallers (*n*=27)*	*Non-fallers (*n*=60)*	
*Dopaminergic medication*			
LEU (mg)	734.3 (1616.7)	330.8 (1700)	0.009
Levodopa	22/27 (81%)	36/60 (60%)	0.05
Dopamine agonist	17/27 (46%)	32/60 (53%)	0.40
MAOB inhibitor	3/27 (11%)	8/60 (13%)	0.77
COMT inhibitor	10/27 (37%)	14/60 (23%)	0.19
Amantadine	7/27 (26%)	4/60 (7%)	0.01
			
*Other medication*			
Antidepressants	10/27 (37%)	4/60 (7%)	<0.001
Neuroleptics	11/27 (41%)	5/60 (8%)	<0.001
Sleep medication	2/27 (7%)	3/60 (5%)	0.66

Abbreviation: LEU, Levodopa-equivalent unit.

**Table 3:** Forward conditional logistic regression for prediction of fallers

*Predictive factor*	*Odds ratio (95% CI)*	P*-value*
SCOPA-Autonomic Cardiovascular subscale	1.935 (1.18–3.16)	0.009
Antidepressant use	6.549 (1.59–26.99)	0.008
Presence of REM sleep behavior disorder	3.701 (1.16–11.77)	0.03

Abbreviations: CI, confidence interval; SCOPA, Scales for Outcome in Parkinson’s.

Variables in the equation: Antidepressants, Neuroleptics, Dyskinesia, SCOPA-COG-executive function, SCOPA-AUT-Cardiovascular, PPRS, RBD, Total PSQI score, SCOPA-AUT Pupillomotor, SCOPA-AUT Gastrointestinal, LEU, Duration of diagnosis.
